# SnoRD126 promotes the proliferation of hepatocellular carcinoma cells through transcriptional regulation of FGFR2 activation in combination with hnRNPK

**DOI:** 10.18632/aging.203014

**Published:** 2021-04-23

**Authors:** Weiqi Xu, Yu Wu, Xianlong Fang, Yuxin Zhang, Ning Cai, Jingyuan Wen, Jingyu Liao, Bixiang Zhang, Xiaoping Chen, Liang Chu

**Affiliations:** 1 Hepatic Surgery Center, Tongji Hospital, Tongji Medical College, Huazhong University of Science and Technology, Wuhan, China; 2 Clinical Medical Research Center of Hepatic Surgery in Hubei Province, Wuhan, China; 3 State Key Laboratory of Cell Biology, Shanghai Institute of Biochemistry and Cell Biology, Chinese Academy of Sciences, Shanghai, China

**Keywords:** snoRD126, HCC, small nucleolar RNA, PI3K-AKT

## Abstract

Liver cancer is the sixth most common malignancy and the fourth leading cause of cancer-related death worldwide. Hepatocellular carcinoma (HCC) is the primary type of liver cancer. Small nucleolar RNA (snoRNA) dysfunctions have been associated with cancer development. SnoRD126 is an orphan C/D box snoRNA. How snoRD126 activates the PI3K-AKT pathway, and which domain of snoRD126 exerts its oncogenic function was heretofore completely unknown. Here, we demonstrate that snoRD126 binds to hnRNPK protein to regulate FGFR2 expression and activate the PI3K-AKT pathway. Importantly, we identified the critical domain of snoRD126 responsible for its cancer-promoting functions. Our study further confirms the role of snoRD126 in the progression of HCC and suggests that knockdown snoRD126 may be of potential value as a novel therapeutic approach for the treatment of HCC.

## INTRODUCTION

Liver cancer is the sixth most common malignancy and the fourth leading cause of cancer-related death worldwide [1]. Hepatocellular carcinoma (HCC) is the primary type of liver cancer, accounting for 75-85% of all liver cancer cases. Hepatocellular carcinoma (HCC) is a leading cause of cancer-related death worldwide, especially in Asia and sub-Saharan Africa [2]. The principal risk factors for the development HCC are hepatitis B virus (HBV), hepatitis C virus (HCV), alcoholism, and aflatoxin contamination in food [3]. Although the diagnosis and treatment of HCC have been improved in recent decades, the survival time of HCC patients remains one of the shortest among all cancers [4], which is exacerbated by its complicated pathogenesis.

Small nucleolar RNAs (snoRNAs) are intermediate-length non-coding RNAs, typically ranging from 60nt to 300nt, and are hosted in the introns of protein-coding and non-protein-coding genes [5]. They are primarily classified into C/D box and H/ACA box snoRNAs. The C/D box snoRNAs contain evolutionarily conserved C (RUGAUGA) and D (CUGA) box motifs, which are located a few nucleotides away from the 5′ and 3′ ends, respectively. The H/ACA box snoRNAs contain two large hairpin domains that are linked by a conserved H-box motif (ANANNA) and a short ACA tail at the 3′ end [6]. Many C/D snoRNAs contain a less-well-conserved duplication of the C and D boxes (referred to as C’ and D’) in the central RNA region [7]. The C and D boxes are frequently brought together to form a hairpin structure by an interaction between the 5’- and 3’-terminal helices [8].

Most non-coding RNAs form ribonucleoprotein (RNP) complexes that recognize targeted nucleic acid sequences via direct base-complementarity interactions, and guide the post-transcriptional modification of pre-rRNAs [9]. The C/D box snoRNAs induce the methylation of pre-rRNAs at the 2’-O-hydroxyl positions, while the H/ACA box snoRNAs direct the site-specific synthesis of ribosomal pseudouridine residues [10]. The resulting modified nucleotides cluster around the universal core regions of rRNAs and are evolutionarily conserved, suggesting that the modifications contribute to essential aspects of ribosomal function [11]. C/D snoRNAs associate with four evolutionarily conserved core snoRNPs, named FBL (fibrillarin, methyltransferase) [7], NOP56 (nucleolar protein 56) [12], NOP58 (nucleolar protein 58) [13] and NHP2L1 (non-histone chromosome protein 2-like 1) [14]. They typically promote the 2'-O-ribose methylation of sites located 5 nucleotides upstream of the D or D’ box. In vertebrates, the C and D box motifs are required for binding of ribonucleoproteins, including the essential nucleolar protein methyltransferase, fibrillarin, while the C' and D' boxes are necessary for guided methylation [15].

SnoRD126 is a C/D box snoRNA, located in the intron of the host gene cyclin B1 interacting protein 1 (ccnb1ip1, also known as HEI10), which is a member of the E3 ubiquitin ligase family and regulates the cell cycle. We previously found that snoRD126 was significantly overexpressed in HCC and CRC tissues [16]. Furthermore, snoRD126 was found to increase the proliferation of HCC and CRC cells *in vitro*, as well as the growth of xenograft tumors in nude mice. We demonstrated that snoRD126 contributed to tumorigenesis by activating the PI3K-AKT signaling pathway via the upregulation of fibroblast growth factor receptor 2 (FGFR2). However, the snoRD126 associated proteins and structural elements required for the function of snoRD126 remain poorly understood, and few studies focused on targeting snoRNAs. In the present study, we investigated the therapeutic potential of targeting snoRD126 in HCC.

## RESULTS

### SnoRD126 activates the PI3K-AKT pathway

**Figure 1:**
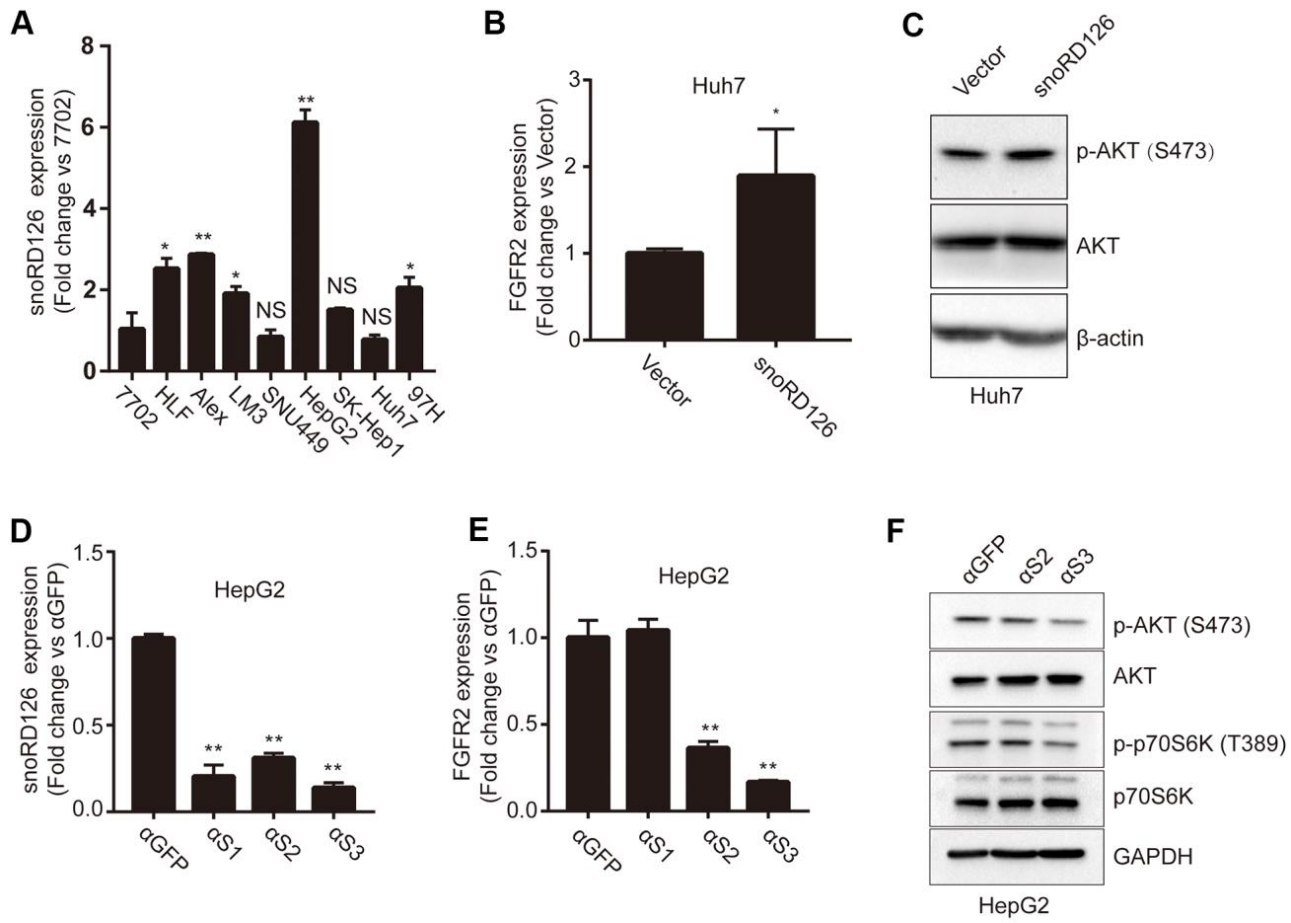
**SnoRD126 regulates the phosphorylation of AKT and the expression of FGFR2.** (**A**) qRT-PCR assay for snoRD126 expression in normal human hepatocyte 7702 cells and HCC cell lines. (**B**) qRT-PCR assay for FGFR2 expression in snoRD126-overexpressing Huh7 cells. (**C**) Phosphorylation of AKT was determined by immunoblotting in snoRD126-overexpressing Huh7 cells. (**D**) qRT-PCR assay for snoRD126 knockdown in HepG2 cells treated with ASOs. (**E**) The mRNA levels of FGFR2 in snoRD126-knockdown HepG2 cells as measured by qRT-PCR assay. αS1, αS2, and αS3 are ASOs that target snoRD126. HepG2 cells were treated with 25μM ASO for 24 hours. (**F**) Phosphorylation of AKT was reduced in snoRD126-knockdown HepG2 cells as measured by immunoblotting. The data represent mean ± SD (n = 3). *P < 0.05, **P < 0.01. 7702 cell in (**A**), loading control.

We have previously reported that snoRD126 activates the PI3K-AKT pathway by upregulating FGFR2 [16]. It has also been demonstrated that FGFR2 positively regulates Akt phosphorylation in neurons [17] and Pancreatic Cancer [18], and Akt phosphorylation is reduced when FGFR2 is knocked down. First, we examined the expression levels of snoRD126 in a panel of liver cancer cell lines and the normal human hepatocyte line 7702 (Figure 1A). Five of the HCC cell lines exhibited high expression of snoRD126. Notably, the hepatoblastoma cell line HepG2 had the highest snoRD126 expression. The SNU449 and Huh7 cell lines showed lower expression levels than the other HCC cell lines. Based on these results, we chose the HepG2 and Huh7 cell lines, two widely used liver tumor models, for further analysis. We established stable snoRD126-overexpressing Huh7 cells and snoRD126-knockdown HepG2 cells using antisense oligonucleotides (ASOs). We constructed a Huh7 cell line with stable overexpression of snoRD126 (Huh7-snoRD126), and determined the mRNA levels of FGFR2 in Huh7 cell lines with overexpression of snoRD126 by qRT-PCR. The FGFR2 mRNA levels were significantly higher in Huh7-snoRD126 cells than in the control cells (Huh7-Vector) (Figure 1B). Subsequently, western blot (WB) analysis verified the changes of protein levels. We found that overexpression of snoRD126 increased AKT phosphorylation (Figure 1C). In order to reduce the expression of snoRD126, we synthesized three siRNAs targeting different sequences of snoRD126. After transfection of HepG2 (Supplementary Figure 1A) and Huh7 (Supplementary Figure 1B) cells, the qRT-PCR assay was used to detect the expression of snoRD126. We found that these siRNAs did not knock down the expression of snoRD126. We then performed the nucleo-cytoplasmic separation test and found that 126 was expressed at higher levels in the nucleoli then in the cytoplasm or nucleoplasm (Supplementary Figure 2A). At the same time, we also conducted a fluorescence *in situ* hybridization (FISH) assay, and the results indicated that snoRD126 was localized in the nucleus (Supplementary Figure 2B). It is possible that the siRNA could not effectively knock down snoRD126 because it is mainly found in the nucleus, while siRNAs target RNAs in the cytoplasm. Consequently, we next used ASOs, which could indeed knock down the expression of snoRD126 (Figure 1D). After treating HepG2 cells with 25μM ASOs for 24h, the transcription level of FGFR2 also decreased according to qRT-PCR (Figure 1E). However, the FGFR2 expression level did not decrease after treatment with αS1. We suspected that αS1 might be insufficiently specific to knock down snoRD126 and did not use it in the subsequent experiments. Both phosphorylated p70S6K and phosphorylated AKT levels decreased in HepG2 cells following ASO treatment (Figure 1F). All these results indicated that snoRD126 enhanced FGFR2 transcription and activated the PI3K-AKT pathway.

### The C' and D boxes are crucial for the interaction between snoRD126 and hnRNPK

**Figure 2:**
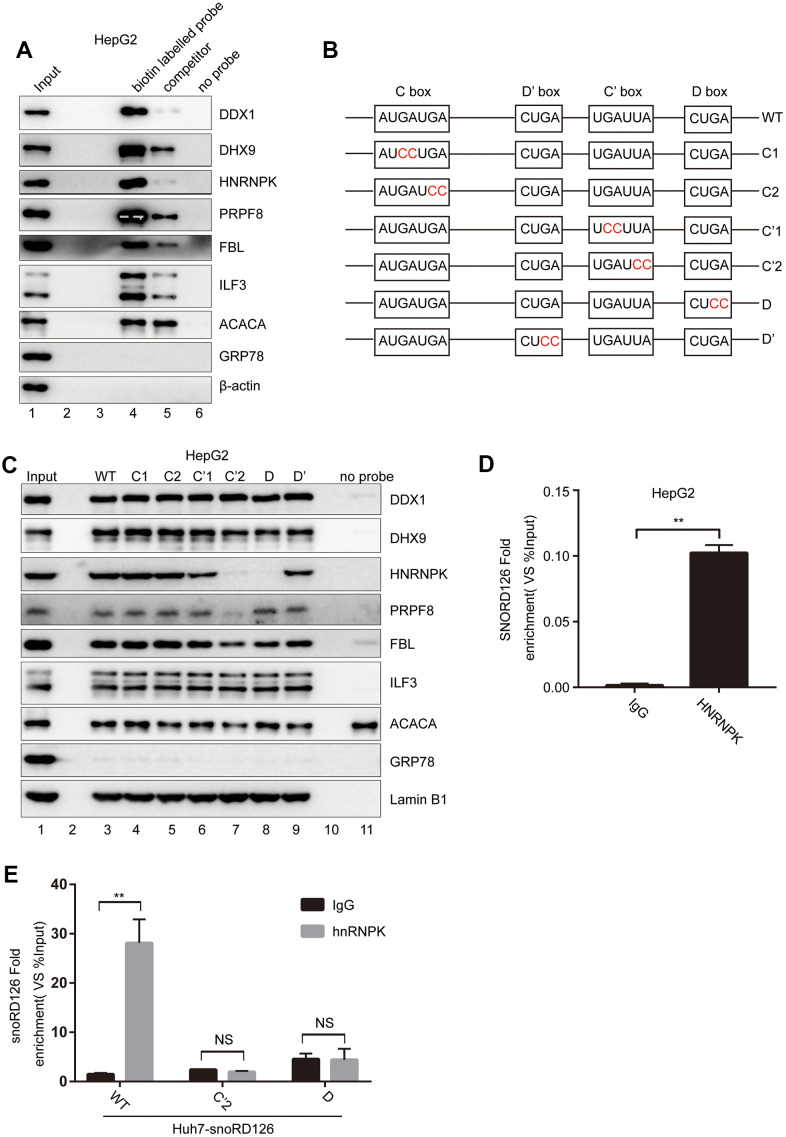
**SnoRD126 combines with hnRNPK.** (**A**) Confirmation of snoRD126-binding proteins by RNA pull-down followed by immunoblotting. Lanes 1, 10% of the cell extracts used in the pull-down assay. Lane 4, biotin-labelled snoRD126 probe. Lane 5, biotin-labelled and biotin-unlabelled mixed snoRD126 probe used as a competitor. Lane 6, without RNA probe. (**B**) Schematic diagrams of the wild-type (WT) snoRD126 and the mutants with double nucleic acid base mutations in its four conserved motifs. (**C**) RNA pull-down followed by immunoblotting using biotin-labelled WT (lane 3) and mutants of snoRD126 (lane 4-9). Lanes 1, 10% of the cell extracts used in the pull-down assay. Lane 11, without RNA probe. (**D**, **E**) Confirmation of snoRD126 presence in a hnRNPK ribonucleoprotein complex by RIP assay followed by a qRT-PCR assay using (**D**) HepG2 cells transfected with pcDNA3.1^+^ plasmid expressing an N-terminal 3xFlag-tagged hnRNPK (pcDNA3.1^+^-3xFlag-hnRNPK); (**E**) Huh7 cells stably expressing snoRD126, SNORD126 C’2 and SNORD126 D mutants. The data represent mean ± SD (n = 3). **P < 0.01. NS, not significant.

To further investigate how snoRD126 upregulates the expression of FGFR2 to activate the PI3K-Akt pathway, we performed a pull-down experiment with biotin-labeled snoRD126, after which the purified protein complex was analyzed by mass spectrometry (Supplementary Table 1). The RNA pull-down experiment was repeated, followed by western blot analysis to verify the candidate snoRD126-binding proteins (Figure 2A). It was found that the binding strength of these proteins to the biotin-labelled snoRD126 probe was significantly greater than that of the biotin-labelled and biotin-unlabelled mixed snoRD126 probe (competitor), or the magnetic beads (no probe). Since snoRD126 is a C/D box snoRNA, it is generally believed that its functional area lies within the C or D box. Accordingly, point mutations were made in the four domains (C box, D box, C' box and D' box) (Figure 2B), and the mutated snoRD126 probes were used in the pull-down experiment (Figure 2C). We found that the hnRNPK and PRPF8 protein bands were weaker when the C'2 and D domains of snoRD126 were mutated. These results suggested that C'2 and D are probably the functional domains, which affect the binding of snoRD126 to hnRNPK and PRPF8 proteins. The hnRNPK plasmid (pcDNA3.1+hnRNPK) was transiently introduced into HepG2 and Huh7 cell lines with stable overexpression of wild type (WT) snoRD126 or the C'2 and D mutations, respectively. Then, the RIP experiment was conducted using an hnRNPK antibody to determine the amount of snoRD126 or its mutant variants in the pull-downs by qRT-PCR (Figure 2D, 2E). We detected specific binding of hnRNPK and snoRD126, which was substantially reduced in the snoRD126 mutants (Figure 2E). Collectively, our data suggested that snoRD126 works by binding to hnRNPK protein through the structural C' and D domains.

### The C' and D boxes are crucial for the function of snoRD126

**Figure 3:**
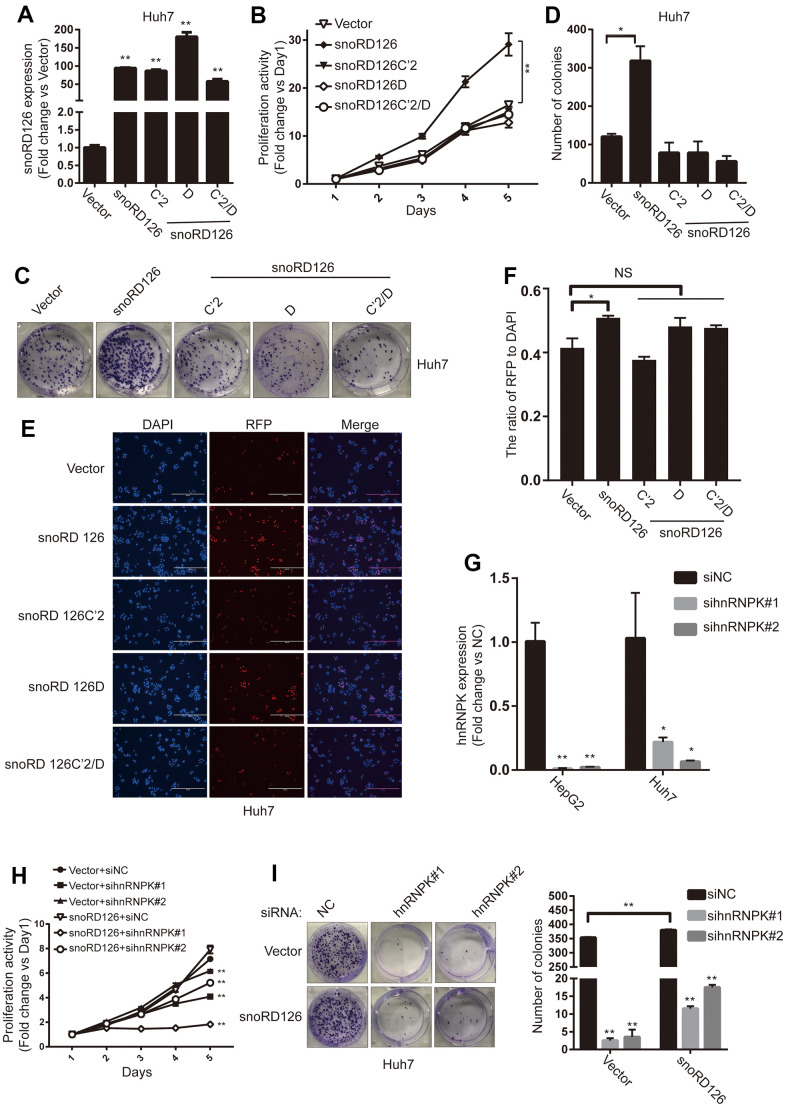
**C' box and D box are crucial for snoRD126 to promote cell proliferation.** (**A**) qRT-PCR assay for snoRD126 expression in Huh7 cells with snoRD126 WT or mutants-overexpressing. (**B**) CCK8 assay performed in Huh7 cells with snoRD126 WT or mutants-overexpression. (**C**, **D**) Representative colony formation images and quantification of Huh7 cells with snoRD126 WT or mutants-overexpression. (**E**, **F**) Representative EdU images and quantification of Huh7 cells with snoRD126 WT or mutants-overexpression. Scale bar, 400um. (**G**) qRT-PCR assay for hnRNPK expression in HepG2 cells and snoRD126-overexpressing Huh7 cells with hnRNPK siRNA treatment. (**H**) CCK8 assays performed in snoRD126- or vector-overexpressed Huh7 cells with hnRNPK siRNA treatment. (**I**) Representative colony formation images and quantification of snoRD126- or vector-overexpressed Huh7 cells with hnRNPK siRNA treatment. The data represent mean ± SD (n = 3). *P < 0.05, **P < 0.01. NS, not significant.

We next constructed snoRD126C'2/D-overexpressing Huh7 cells to verify whether combined C'2 and D mutations would have a full impact on the functionality of snoRD126 (Figure 3A). The CCK8 assay was performed to evaluate if the overexpression of snoRD126 WT, but not the C'2 or D domain mutants, promoted cell proliferation (Figure 3B). Moreover, the results were confirmed by colony formation and EdU incorporation assays (Figure 3C–3F). Additionally, qRT-PCR was performed in the HepG2 and Huh7 to detect the expression of hnRNPK after transfection with hnRNPK siRNAs (Figure 3G). The results suggested that both sequences are valid, and that hnRNPK can be efficiently knocked down. The hnRNPK siRNAs were then introduced into snoRD126-overexpressing Huh7 cells. The proliferation-promoting effect of snoRD126 overexpression was significantly inhibited by hnRNPK knockdown (Figure 3H, 3I). These results suggested that the C' and D boxes are the key structural domains of snoRD126 that promote cell proliferation, whereby snoRD126 may play a role in promoting cell proliferation by binding to hnRNPK.

### SnoRD126 upregulates FGFR2 expression in combination with hnRNPK

**Figure 4:**
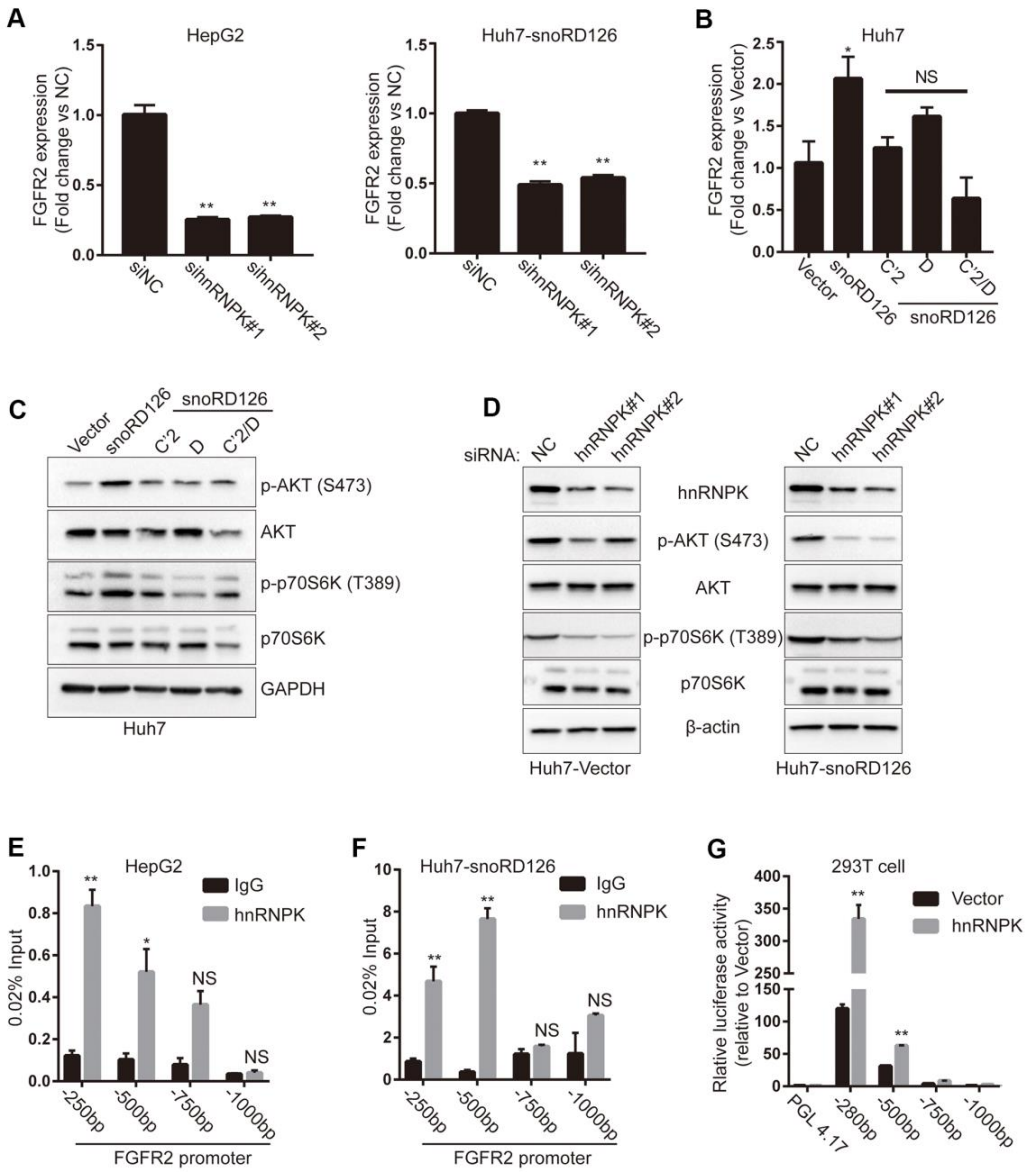
**SnoRD126 combining with hnRNPK to up-regulate FGFR2.** (**A**) qRT-PCR assay for FGFR2 expression in HepG2 cell and snoRD126-overexpressing Huh7 cells after transfected with hnRNPK siRNAs. (**B**) qRT-PCR assay for FGFR2 expression in Huh7 cells with snoRD126 WT or mutants-overexpressing. (**C**) Overexpression of snoRD126 rather than the mutants activated phosphorylation of AKT and p70S6K in Huh7 cells as measured by immunoblotting. (**D**) Immunoblotting was performed for the indicated proteins in snoRD126- or vector-overexpressed Huh7 cells with hnRNPK siRNA treatment. (**E**, **F**) ChIP assay using hnRNPK antibody followed by qRT-PCR assay for hnRNPK occupancy at FGFR2 promoter region in (**E**) HepG2 and (**F**) snoRD126-overexpressed Huh7 cells. Four primer pairs were used to assess the occupancy at every 250 bp upstream of FGFR2 transcription start site (TSS). (**G**) Luciferase reporter assays for FGFR2 promoter activity after transiently transfected hnRNPK in HEK-293 cells, mean ± SD, **P<0.01, NS, not significant. GAPDH and β-actin in (**C**, **D**), loading control.

First, we used qRT-PCR to quantify the expression of FGFR2 in HepG2 cells and snoRD126-overexpressing Huh7 cells after transfection with hnRNPK siRNAs. The results suggested that knockdown of hnRNPK decreased the mRNA expression of FGFR2 (Figure 4A). We then used qRT-PCR to examine FGFR2 expression after stably overexpressing snoRD126 and its mutants in Huh7 cells (Figure 4B). The results indicated that FGFR2 transcription was elevated in the Huh7-snoRD126 cell line compared to the Huh7-Vector cells, but not in the cells transfected with the mutant constructs (Huh7- snoRD126C’2, Huh7- snoRD126D and Huh7-snoRD126C’2/D). Western blot analysis indicated that phosphorylation of AKT and p70S6K was not induced in the mutant-transfected cells (Figure 4C). Knockdown of hnRNPK in snoRD126-overexpressing Huh7 cells restored the activation of the PI3K-AKT pathway by snoRD126 (Figure 4D). Considering the interaction between snoRD126 and hnRNPK, as well as the regulatory effect of snoRD126 mutants and hnRNPK on FGFR2, we suspected that the increase of FGFR2 transcription may be due to transcriptional regulation of FGFR2 by a combination of snoRD126 and hnRNPK. To understand the mechanism of FGFR2 regulation by the snoRD126/hnRNPK complex, we first investigated the interaction of the snoRD126/hnRNPK complex with the proximal promoters of FGFR2 genes by ChIP experiments in HepG2 and Huh7-snoRD126 cells (Figure 4E, 4F). Our analysis revealed a significant enrichment of hnRNPK at the FGFR2 promoter (0 to -500 bp relative to the transcription start site). We then investigated the role of hnRNPK in the transcriptional regulation of FGFR2 using a luciferase reporter assay in HEK-293 cells (Figure 4G), and the data were consistent with the ChIP results. HEK-293 cells (more widely known as the Human Embryonic Kidney 293 cells), was derived in 1973 by exposing the human primary embryonic kidney cell culture of an aborted embryo to the mechanically sheared DNA of adenovirus type 5 (AD5) [19].

### The snoRD126 mutants did not affect tumor growth *in vivo*

**Figure 5:**
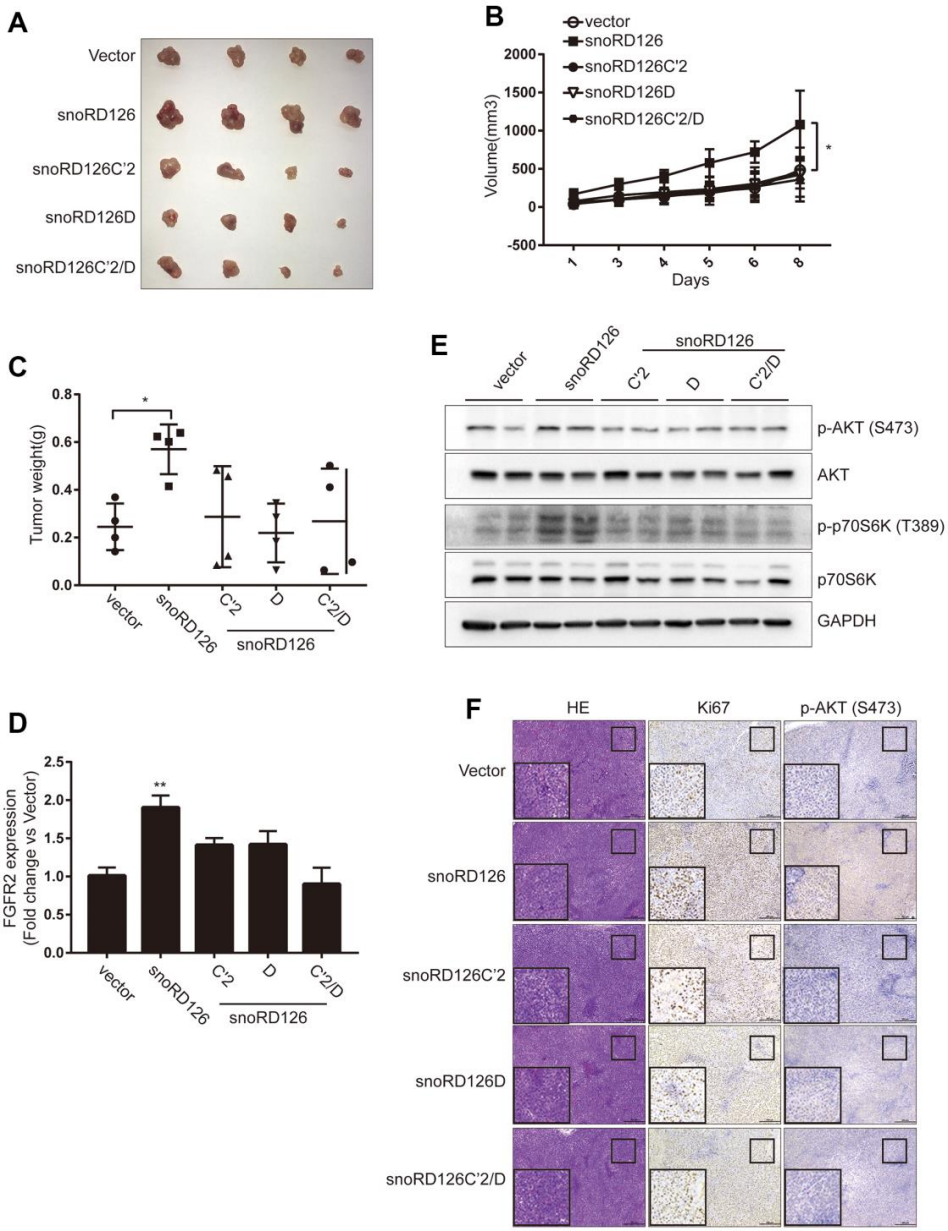
**SnoRD126 mutants did not change the rate of tumour growth *in vivo*.** (**A**) The xenograft tumours photograph after stably overexpressing snoRD126 and its mutants in Huh7 cells. (**B**) Overexpressing snoRD126, not the mutants, increased tumour volume. (**C**) Overexpressing snoRD126, not the mutants, increased tumour weights. (**D**) WB assay was used to analyze the changes of the indicated protein levels in tumours. (**E**) qRT-PCR assay for FGFR2 expression in tumours. (**F**) Immunohistochemistry showing snoRD126 overexpressing led to an increase of Ki67 protein and phosphorylation of AKT levels rather than its mutants. mean ± SD, *P<0.05, **P<0.01.

To determine whether these results were reproducible *in vivo*, we constructed a subcutaneous xenograft model of HCC in nude mice. Huh7-snoRD126 cells exhibited increased growth and tumor weight of subcutaneous xenografts compared to the Huh7-Vector group (Figure 5A–5C). However, there were no significant changes in the size, volume and weight of the subcutaneous xenograft tumors in the Huh7-mutant groups (Huh7-snoRD126C’2, Huh7-snoRD126D and Huh7-snoRD126C’2/D) compared to the Huh7-Vector group, confirming the *in vitro* results. The intratumoral transcription levels of FGFR2 were also determined by qRT-PCR (Figure 5D). The phosphorylation levels of proteins in the PI3K-AKT pathway inside the tumors were also determined by WB analysis, and there was no increase in AKT phosphorylation in the mutant groups compared to the control group (Figure 5E). Finally, the subcutaneous tumors were fixed with paraformaldehyde and then stained with HE, as well as antibodies against Ki67, and p-AKT (S473) (Figure 5F). These results corroborated the initial *in vitro* findings that the tumor-promoting effect of snoRD126 was abrogated if its C' and D domains were mutated.

### ASOs targeting snoRD126 inhibited tumor growth *in vivo*

**Figure 6:**
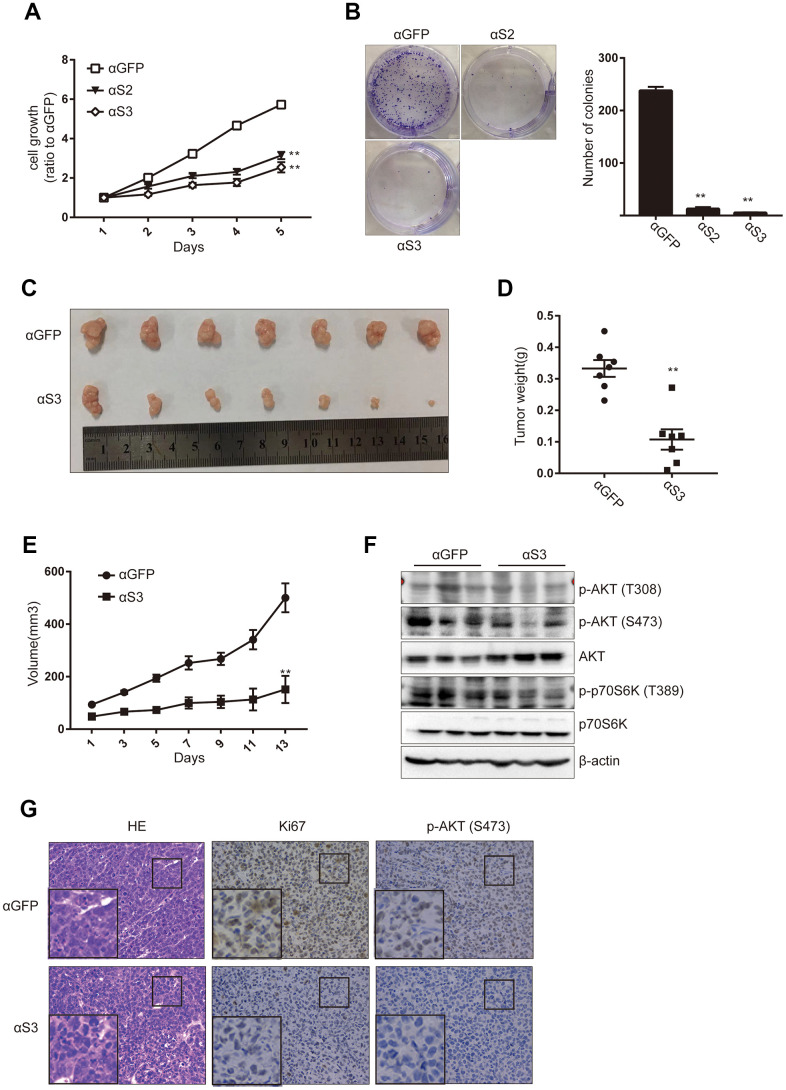
**ASOs targeting snoRD126 inhibit tumour growth *in vivo*.** (**A**) CCK8 assay was performed after transiently transfected HepG2 cells with snoRD126 ASOs. (**B**) Colony formation assay was performed in HepG2 cells after transfected with snoRD126 ASOs. Quantification of the colonies normalized to αGFP group is presented. (**C**) The xenograft tumours photograph after injecting snoRD126 ASOs into subcutaneous tumours. (**D**) Tumour weight is presented. (**E**) Tumour volume is presented. (**F**) WB assay was used to analyze the changes of the indicated protein levels in tumours. (**G**) Immunohistochemistry showing intratumoral injection of aS3 led to an increase of Ki67 protein and phosphorylation of AKT levels. Mean ± SD. ** p < 0.01.

The metabolic activity of HepG2 cells was detected using the CCK8 assay following 24h after ASO treatment (Figure 6A). The colony formation assay was also performed to detect the effects of ASO treatment on cell proliferation (Figure 6B). Cell growth and proliferation were significantly inhibited after knockdown of snoRD126 compared with the control group (αGFP). To determine whether these results were reproducible *in vivo*, we used a subcutaneous xenograft model of HCC in nude mice based on HepG2 cells. When the tumor grew to 32mm3, ASOs were injected into the tumors every other day for two weeks. After that, the mice were sacrificed to extract the subcutaneous tumors. The tumors were significantly smaller in the αS3 group than in the αGFP control group (Figure 6C). The weight (Figure 6D) and volume (Figure 6E) of the subcutaneous tumors exhibited consistent trends. WB analysis demonstrated that the activation of the PI3K-AKT pathway was significantly inhibited in the αS3 group compared with the αGFP group (Figure 6F). Finally, the subcutaneous tumors were fixed with paraformaldehyde, and then stained with HE, as well as antibodies against Ki67 and p-AKT (S473) (Figure 6G). These results confirmed that ASOs that inhibit the expression of snoRD126 can also inhibit the growth and proliferation of tumors *in vivo*.

### SnoRD12 is highly expressed in HCC patient samples and is correlated with poor prognosis

**Figure 7:**
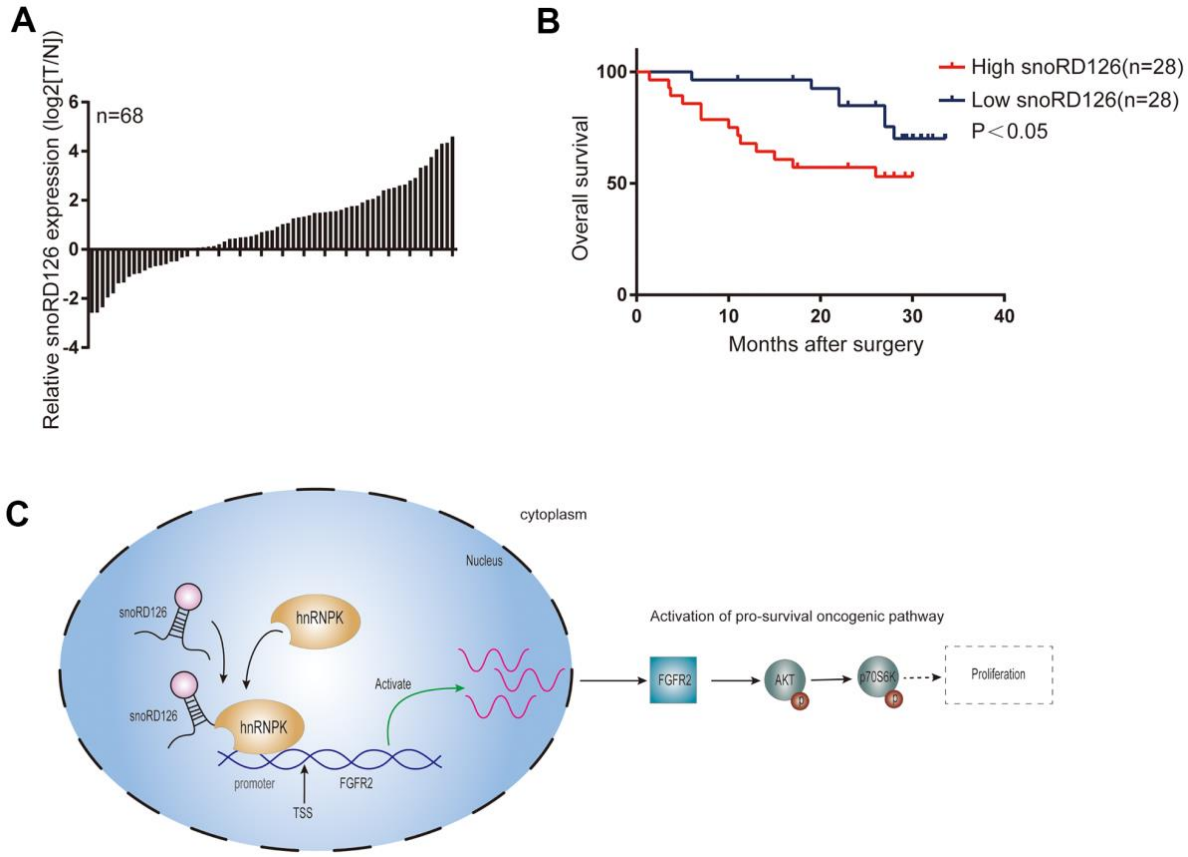
**SnoRD12 is highly expressed in HCC patient samples and represents poor prognosis.** (**A**) qRT-PCR assay detected snoRD126 expression in 68 pairs of HCC and adjacent nontumorous liver tissues. snoRD126 in HCC tissues were quantified and are shown in the bar chart after being normalized to their own adjacent nontumorous liver tissues. (**B**) Kaplan-Meier’s analysis of the correlation between snoRD126 expression and the overall survival of HCC patients. (**C**) Schematic model according to the results of this study.

After confirming the initial *in vitro* findings in an animal model *in vivo*, we finally examined the expression of snoRD126 in 68 pairs of HCC tumors (Table 1) and adjacent non-cancerous tissues by qRT-PCR (Figure 7A). The average expression level of snoRD126 was significantly higher in HCC tissues than in adjacent non-cancerous tissues. We then explored the correlation between snoRD126 expression and patient outcomes using the GEPIA [20] and Kaplan Meier-plotter [21] websites. Higher expression of snoRD126 predicted shorter overall survival (OS) in both patient cohorts. Thus, snoRD126 was highly expressed in HCC and predicted poor clinical outcomes.

**Table 1:** Characteristics about 68 samples used for survival analysis.

**Clinicopathologicalvariables**	**NumberN=68**	**Percentage**
**Gender**		
Male	55	80.88
Female	13	19.12
**Age**		
≤50	33	48.53
>50	35	51.47
**AFP (ug/L)**		
≤20	18	26.47
>20	50	73.53
**HBV**		
Negative	8	11.76
Positive	60	88.24
**HCV**		
Negative	67	98.53
Positive	1	1.47
**Tumor size (cm)**		
≤5	36	52.94
>5	32	47.06
**Vascular invasion**		
No	50	73.53
Yes	18	26.47
**Differentiation**		
High	9	13.23
Moderately	31	45.6
Low	28	41.17
**Distant metastasis**		
No	60	88.24
Yes	8	11.76
**TNM stage**		
T1	14	20.59
T2-T3-T4	54	79.41
**BCLC stage**		
0+A	42	61.76
B+C	26	38.24
**Adjuvant TACE**		
No	52	76.47
Yes	16	23.53

## DISCUSSION

SnoRD126, a relatively recently discovered non-coding RNA comprising 77 nucleotides, plays essential roles in regulating gene expression to promote the progression of HCC [16]. However, recent evidence indicates that snoRD126 also has other functions. For example, it promotes normal adipogenesis [22] and hepatitis C virus (HCV) infection by activating the PI3K-AKT pathway [23]. In our previous work, we demonstrated that snoRD126 promotes the progression of hepatocellular and colorectal cancer by upregulating FGFR2 to activate the PI3K-AKT pathway. However, it was unclear if snoRD126 is essential for the tumorigenesis of HCC. Accordingly, it was unknown how snoRD126 activates the PI3K-AKT pathway and which domain of snoRD126, which is a C/D box snoRNA, exerts its pro-tumorigenic function. In this study, we discovered that snoRD126 is highly expressed in HCC, and that it predicts poor clinical outcomes. We demonstrated that snoRD126 binds to hnRNPK protein to regulate FGFR2 expression, thereby activating the PI3K-AKT pathway (Figure 7C). Further cell culture and mouse xenograft models demonstrated that snoRD126 possesses oncogenic properties, driving the progression of HCC. Accordingly, ASOs that can inhibit the expression of snoRD126 also inhibited the growth of HCC cells. The discovery of such a snoRNA provides a promising new target for therapeutic interventions against HCC.

HnRNPK belongs to the heterogeneous nuclear ribonucleoprotein family [24]. As an evolutionarily conserved nucleocytoplasmic shuttling protein, hnRNPK participates in chromatin remodeling and transcriptional regulation, as well as influencing the splicing, stability, and translation of mRNAs [25]. HnRNPK has been shown to regulate the expression of multiple genes, including c-Src [26], c-Myc [27], thymidine kinase 1 [28], eukaryotic translation initiation factor 4E [29], androgen receptor [30], and p53 target genes [31]. Furthermore, hnRNPK was also found to enhance the translation of FGFR2 mRNA [32], and there is evidence that FGFR2 activates the PI3K-AKT pathway [33]. There is also considerable evidence that hnRNPK is closely related to the progression of HCC [34]. In this study, we found that snoRD126 binds to hnRNPK and regulates FGFR2 expression, promoting the development of liver cancer.

In summary, we demonstrated that snoRD126 binds to hnRNPK protein and upregulates FGFR2 transcription, thereby activating the PI3K-Akt pathway to promote the progression of HCC. Importantly, we identified the critical domains of snoRD126 that mediate its cancer-promoting effects. However, we only demonstrated that the C' and D domains are essential for the binding of snoRD126 to hnRNPK and its function. Additional studies are required to explore how the C' and D domains of snoRD126 interact with hnRNPK protein. It remains unknown whether the conserved C’ and D domains are critical for the other C/D box snoRNAs, and this question merits further exploration. The present study suggests that knockdown of snoRD126 by ASOs may be a potential therapeutic strategy for HCC.

## MATERIALS AND METHODS

### Patients and tissue specimens

Human tumour and adjacent non-tumour tissues were collected from HCC patients underwent hepatectomy at the Hepatic Surgery Center, Tongji Hospital of Huazhong University of Science and Technology (HUST) (Wuhan, China). All procedures were conducted according to the Declaration of Helsinki Principles and approved by the Ethics Committee of Tongji Hospital, HUST. All patients received written informed consent for data analysis before surgery.

### Xenograft study

All animal studies were performed in compliance with the National Institutes of Health guidelines (NIH publication 86-23, revised 1985). All animal experiments were approved by the Committee on the Ethics of Animal Experiments of Tongji Medical College, Huazhong University of Science and Technology. Male Balb/c athymic nude mice (5-week-old) were used and are bred in SPF animal house. The mice were randomly divided into the indicated groups (5–7 mice/group) before inoculation. 2×10^6^ tumour cells for subcutaneous injection were suspended in 100 μL of serum-free DMEM and then injected subcutaneously in the dorsal region of nude mice. Tumour development in the mice was observed every other day. All mice were sacrificed at a defined endpoint. Tumours were removed, photographed, measured, and weighed. The average volume and weight of the tumours were calculated. In the therapeutic experiments, when tumours grew to about 32mm^3^, ASO (50nM, 20ul per mouse) was injected every other day for two weeks.

### Cell lines and culture

HCC cell line Huh7 was obtained from the State Key Laboratory of Cell Biology, Institute of Biochemistry and Cell Biology, Shanghai Institutes for Biological Sciences, Chinese Academy of Sciences, Shanghai, China. HepG2 and 293T cells were purchased from the China Center for Type Culture Collection (CCTCC, Wuhan, China). All cell lines were maintained in Dulbecco Modified Eagle Medium (DMEM) (Hyclone, UT, USA) supplemented with 10% fetal bovine serum (FBS) (Gibico) at 37° C in a 5% CO2 cell incubator.

### Plasmids

To establish snoRD126, snoRD126C’2, snoRD126D and snoRD126C’2/D overexpressing cell lines, the human snoRD126 sequence and its mutants were cloned into the pLKO.1 vector (pLKO.1 puro, plasmid #8453, Addgene). Viral packaging and transduction were performed as previously described [35].

### Cell counting kit 8 assay and EdU incorporation assay

CCK8 (Dojindo, Kumamoto, Japan) was performed according to the protocol provided by the manufacturer. Optical density (OD) was measure by Universal Microplate Reader ELx 800 (BIO-TEK, USA) at 450 nm wavelength. After extracting blank value, the results of the formula (experimental OD value/control OD value) were used for indication of the cell viability. We seeded HCC cells (4000 cells/well) into 96-well plates and cultured overnight for EdU incorporation assay by using Cell-Light™ EdU Apollo567 *In Vitro* Imaging Kit (Ribobio, Guangzhou, China) following the protocol provided by the manufacturer. We captured representative images with EVOS FL auto imaging system (Life Technologies, USA) and counted positive cells by Image Pro. Plus version 6.0.

### Transfection and luciferase reporter assays

Small interfering RNAs (siRNAs), si-control (NC) (sequences in Table 2) and antisense oligonucleotides (ASOs) (sequences in Table 3) were purchased from Ribobio (Guangzhou, China). Lipofectamine 3000 (Invitrogen, Carlsbad, CA, USA) was used for the transient transfection of small RNA oligos, ASOs and plasmids according to the manufacturer’s instructions. siRNA target sequences are listed in Table 2. We used a dual-specific luciferase assay kit (Promega) to perform luciferase assays according to the manufacturer's protocols after transfection. We repeated reporter assay three times.

**Table 2:** The primers and the sequence of siRNA used in this study.

siRNA	siHNRNPK#1	5‘-GCAUAAAGAUCAUCCUUGA-3’
	siHNRNPK#2	5‘-CCAACAUUCCUCUGCUUCA-3’
	siNC	5‘-UUCUCCGAACGUGUCACGU-3’
Primers	Homo-HNRNPK-forward	5’-CAATGGTGAATTTGGTAAACGCC-3’
	Homo-HNRNPK-reverse	5‘-GTAGTCTGTACGGAGAGCCTTA-3’
	Homo-GAPDH-forward	5’-CTGGGCTACACTGAGCACC-3’
	Homo-GAPDH-reverse	5‘-AAGTGGTCGTTGAGGGCAATG-3’
	Homo-U6-forward	5’-TCGCTTCGGCAGCACATATAC-3’
	Homo-U6-reverse	5‘-GCGTGTCATCCTTGCGCAG-3’
	Homo-126(99)- forward	5’-TCAGTCATTTACAGTTTGCCAT-3’
	Homo-126(99)- reverse	5‘-CCTAGCTTTAGTCTGCTCAGAG-3’
	Homo-126(77)- forward	5’-AGTTTGCCATGATGAAATGC-3’
	Homo-126(77)- reverse	5‘-CTCAGAGCATGTGTTTAATCAG-3’
	Homo-FGFR2-forward	5’-ACACAGGATGGGCCTCTCTA-3’
	Homo-FGFR2-reverse	5‘-GCTCCTCAGGAACACGGTTA-3’

**Table 3:** The sequence of three ASOs (αS1, αS2, αS3) and the nonsense sequence with α GFP tag was used as the control group (Ctrl).

**Name**	**Sequence**
Ctrl αS1 αS2 αS3	5’-mU*mC*mA*mC*mC*T*T*C*A*G*C*C*T*C*T*mC*mG*mA*mG*mU-3’ 5’-mG*mA*mU*mC*mA*G*C*T*G*A*A*A*C*A*C*mG*mG*mA*mC*mU*-3’ 5’-mA*mG*mC*mA*mU*G*T*G*T*T*T*A*A*T*C*mA*mG*mG*mC*mU*-3’ 5’-mC*mU*mG*mA*mA*A*C*A*C*G*G*A*C*T*T*mA*mA*mC*mA*mU*-3’

*means phosphorothioate backbone, m means 2’-O-methoxyethyl.

### Quantitative real-time PCR assay

Total RNA was extracted using TRIzol Reagent (TaKaRa Bio Inc, Kusatsu, Shiga, Japan) and reverse transcription was performed using the PrimeScript® RT reagent Kit (TaKaRa Bio Inc, Kusatsu, Shiga, Japan) following the protocol provided by the manufacturer. The quantitative real-time PCR assay was carried out using the CFX96 Touch™ Real-Time PCR Detection System (Bio-Rad, Hercules, CA, USA) using the SYBR Green Supermix kit (TaKaRa Bio Inc, Kusatsu, Shiga, Japan) following the protocol provided by the manufacturer. All of the gene expression levels were normalized to that of the housekeeping gene glyceraldehyde-3-phosphate dehydrogenase (GAPDH), except snoRD126 was normalized to small nuclear RNA U6. All reactions were repeated at least three times independently in triplicate. All primers used are summarized in Table 2.

### Immunohistochemical staining, western blot analysis and colony formation assay

Immunohistochemical staining, western blot analysis and colony formation assay were performed as previously described [35]. The used antibodies were listed in Table 4.

**Table 4:** The antibodies used in this study.

**Antibody**	**Supplier**	**Catalog number**	**Dilution**
AKT	Cell Signaling Technology	4691	WB 1:1000
Phospho AKT (Ser473)	Cell Signaling Technology	4060	WB 1:1000
p70S6K	Cell Signaling Technology	9202	WB 1:1000
Phospho p70S6K (Thr389)	Cell Signaling Technology	9206	WB 1:1000
hnRNPK	abcam	ab70492	WB 1:2000, ChIP- 5μg per assay
GAPDH	Cell Signaling Technology	5174	WB 1:3500
β-actin	Cell Signaling Technology	8457	WB 1:5000

## Supplementary Material

Supplementary Figures

Supplementary Table 1
