# Supplementary material for: SnoRD126 promotes the proliferation of hepatocellular carcinoma cells through transcriptional regulation of FGFR2 activation in combination with hnRNPK
Source: Aging (Albany NY). 2021 Apr 23;13(9):13300–17. doi: 10.18632/aging.203014 (PMC8148486; doi:10.18632/aging.203014)
Supplement: Supplementary Figures [file aging-13-203014-s001.pdf]

SUPPLEMENTARY FIGURES

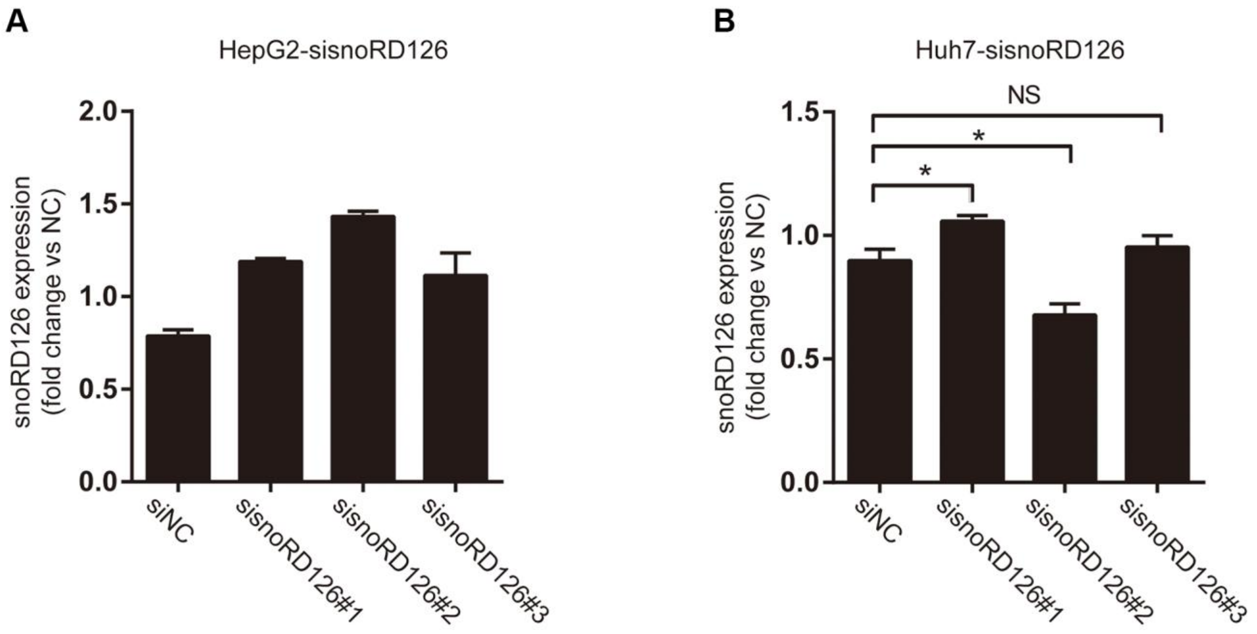

Supplementary Figure 1. (**A**, **B**) qRT-PCR assay for snoRD126 expression in (**A**) HepG2 cells and (**B**) Huh7 cells after treatment with siRNAs targeting snoRD126. Mean ± SD, *P<0.05, NS, not significant.

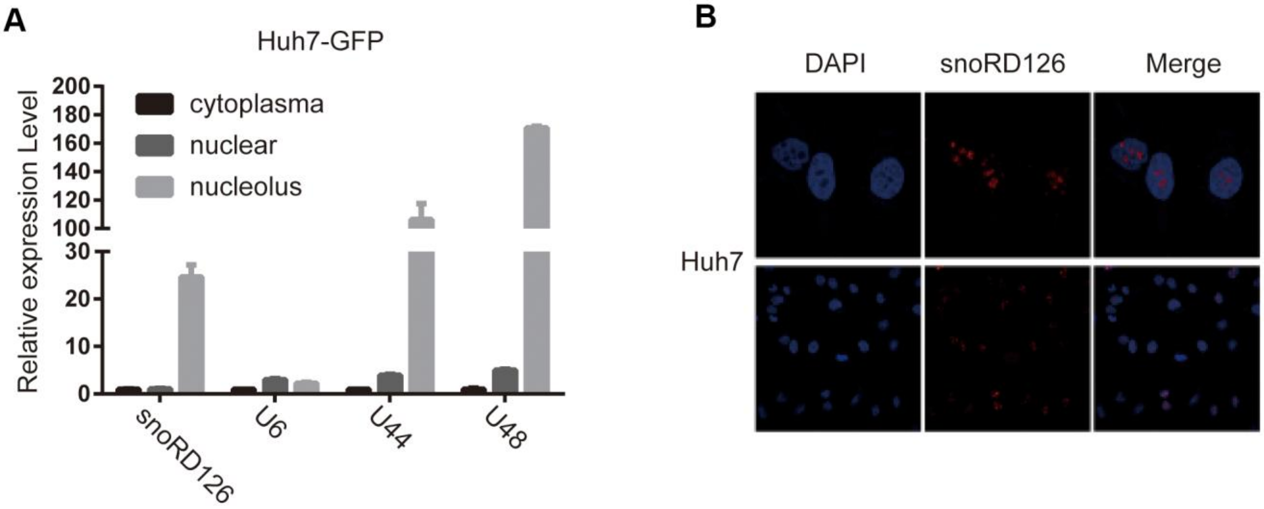

Supplementary Figure 2. (**A**) The nucleo-cytoplasmic separation experiment was performed in Huh7 cells, and the qRT-PCR assay detected the expression of snoRD126, U6, U44, and U48 in the cytoplasm, nuclear and nucleolus segment. (**B**) Fluorescent *in situ* hybridization (FISH) analysis experiment was performed in Huh7 cells.
