# Supplementary material for: SnoRD126 promotes the proliferation of hepatocellular carcinoma cells through transcriptional regulation of FGFR2 activation in combination with hnRNPK
Source: Aging (Albany NY). 2021 Apr 23;13(9):13300–17. doi: 10.18632/aging.203014 (PMC8148486; doi:10.18632/aging.203014)
Supplement: Supplementary Table 1 [file aging-13-203014-s002.docx]

Supplementary Table 1. A pull-down experiment with biotin-labelled snoRD126 from HepG2 cells was performed and analyzed associated proteins by mass spectrometry.

| Bio-function classification | Protein ID | Description | abbreviation |
| --- | --- | --- | --- |
| CRD-mediated complex that promote MYC mRNA stability | Q08211 | ATP-dependent RNA helicase A OS=Homo sapiens GN=DHX9 PE=1 SV=4 | DHX9 |
|  | Q00839 | Heterogeneous nuclear ribonucleoprotein U OS=Homo sapiens GN=HNRNPU PE=1 SV=6 | HNRNPU |
|  | O60506 | Heterogeneous nuclear ribonucleoprotein Q OS=Homo sapiens GN=SYNCRIP PE=1 SV=2 | HNRNPQ |
|  | Q9NZI8 | Insulin-like growth factor 2 mRNA-binding protein 1 OS=Homo sapiens GN=IGF2BP1 PE=1 SV=2 | IGF2BP1 |
| Metabolism | Q00341 | Vigilin OS=Homo sapiens GN=HDLBP PE=1 SV=2 | vigilin |
|  | P11498 | Pyruvate carboxylase, mitochondrial OS=Homo sapiens GN=PC PE=1 SV=2 | PC |
|  | Q12906 | Interleukin enhancer-binding factor 3 OS=Homo sapiens GN=ILF3 PE=1 SV=3 | ILF3 |
|  | Q13085 | Acetyl-CoA carboxylase 1 OS=Homo sapiens GN=ACACA PE=1 SV=2 | ACACA |
|  | P05165 | Propionyl-CoA carboxylase alpha chain, mitochondrial OS=Homo sapiens GN=PCCA PE=1 SV=4 | PCCA |
|  | P11021 | 78 kDa glucose-regulated protein OS=Homo sapiens GN=HSPA5 PE=1 SV=2 | GRP78 |
|  | P51659 | Peroxisomal multifunctional enzyme type 2 OS=Homo sapiens GN=HSD17B4 PE=1 SV=3 | HSD17B4 |
|  | P40939 | Trifunctional enzyme subunit alpha, mitochondrial OS=Homo sapiens GN=HADHA PE=1 SV=2 | HADHA |
|  | P78371 | T-complex protein 1 subunit beta OS=Homo sapiens GN=CCT2 PE=1 SV=4 | CCT2 |
|  | Q9HCC0 | Methylcrotonoyl-CoA carboxylase beta chain, mitochondrial OS=Homo sapiens GN=MCCC2 PE=1 SV=1 | MCCC2 |
|  | Q16822 | Phosphoenolpyruvate carboxykinase [GTP], mitochondrial OS=Homo sapiens GN=PCK2 PE=1 SV=3 | PCK2 |
|  | A8K092 | ATP synthase subunit alpha OS=Homo sapiens GN=ATP5A1 PE=2 SV=1 | ATP5A1 |
|  | P06576 | ATP synthase subunit beta, mitochondrial OS=Homo sapiens GN=ATP5B PE=1 SV=3 | ATP5B |
|  | P00367 | Glutamate dehydrogenase OS=Homo sapiens GN=GLUD1 PE=3 SV=1 | GLUD1 |
|  | P24752 | Acetyl-CoA acetyltransferase, mitochondrial OS=Homo sapiens GN=ACAT1 PE=1 SV=1 | ACAT1 |
|  | P04406 | Glyceraldehyde-3-phosphate dehydrogenase OS=Homo sapiens GN=GAPDH PE=1 SV=3 | GAPDH |
|  | P32322 | Pyrroline-5-carboxylate reductase 1, mitochondrial OS=Homo sapiens GN=PYCR1 PE=1 SV=2 | PYCR1 |
|  | P00338 | L-lactate dehydrogenase A chain OS=Homo sapiens GN=LDHA PE=1 SV=2 | LDHA |
|  | P40926 | Malate dehydrogenase, mitochondrial OS=Homo sapiens GN=MDH2 PE=1 SV=3 | MDH2 |
|  | Q96C36 | Pyrroline-5-carboxylate reductase 2 OS=Homo sapiens GN=PYCR2 PE=1 SV=1 | PYCR2 |
|  | Q13268 | Dehydrogenase/reductase SDR family member 2 OS=Homo sapiens GN=DHRS2 PE=1 SV=3 | DHRS2 |
|  | P49327 | Fatty acid synthase OS=Homo sapiens GN=FASN PE=1 SV=3 | FASN |
| Assembly of the U4/U6-U5 triSNP complex | Q6P2Q9 | Pre-mRNA-processing-splicing factor 8 OS=Homo sapiens GN=PRPF8 PE=1 SV=2 | PRPF8 |
|  | O75643 | U5 small nuclear ribonucleoprotein 200 kDa helicase OS=Homo sapiens GN=SNRNP200 PE=1 SV=2 | SNRNP200 |
|  | B4DK30 | 116 kDa U5 small nuclear ribonucleoprotein component OS=Homo sapiens GN=EFTUD2 PE=2 SV=1 | EFTUD2 |
| Microtubules dynamic | Q9HC35 | Echinoderm microtubule-associated protein-like 4 OS=Homo sapiens GN=EML4 PE=1 SV=3 | EML4 |
|  | P17987 | T-complex protein 1 subunit alpha OS=Homo sapiens GN=TCP1 PE=1 SV=1 | CCT1 |
|  | B4DUR8 | T-complex protein 1 subunit gamma OS=Homo sapiens GN=CCT3 PE=2 SV=1 | CCT3 |
|  | B7Z9L0 | T-complex protein 1 subunit delta OS=Homo sapiens GN=CCT4 PE=2 SV=1 | CCT4 |
|  | B4DEM7 | T-complex protein 1 subunit theta OS=Homo sapiens GN=CCT8 PE=2 SV=1 | CCT8 |
|  | P40227 | T-complex protein 1 subunit zeta OS=Homo sapiens GN=CCT6A PE=1 SV=3 | CCT6A |
|  | E7ENZ3 | T-complex protein 1 subunit epsilon OS=Homo sapiens GN=CCT5 PE=4 SV=1 | CCT5 |
|  | P78371 | T-complex protein 1 subunit beta OS=Homo sapiens GN=CCT2 PE=1 SV=4 | CCT2 |
| Tight junctions and adherens junctions | F5H886 | Tight junction protein ZO-2 OS=Homo sapiens GN=TJP2 PE=4 SV=1 | TJP2 |
|  | G5E9E7 | Tight junction protein 1 (Zona occludens 1), isoform CRA_e OS=Homo sapiens GN=TJP1 PE=4 SV=1 | TJP1 |
| Gene transcription and post-transcription modification | Q00839 | Heterogeneous nuclear ribonucleoprotein U OS=Homo sapiens GN=HNRNPU PE=1 SV=6 | HNRNPU |
|  | P52272 | Heterogeneous nuclear ribonucleoprotein M OS=Homo sapiens GN=HNRNPM PE=1 SV=3 | HNRNPM |
|  | O00571 | ATP-dependent RNA helicase DDX3X OS=Homo sapiens GN=DDX3X PE=1 SV=3 | DDX3X |
|  | Q92841 | Probable ATP-dependent RNA helicase DDX17 OS=Homo sapiens GN=DDX17 PE=1 SV=1 | DDX17 |
|  | O43390 | Heterogeneous nuclear ribonucleoprotein R OS=Homo sapiens GN=HNRNPR PE=1 SV=1 | HNRNPR |
|  | O60506 | Heterogeneous nuclear ribonucleoprotein Q OS=Homo sapiens GN=SYNCRIP PE=1 SV=2 | HNRNPQ |
|  | O15523 | ATP-dependent RNA helicase DDX3Y OS=Homo sapiens GN=DDX3Y PE=1 SV=2 | DDX3Y |
|  | Q92499 | ATP-dependent RNA helicase DDX1 OS=Homo sapiens GN=DDX1 PE=1 SV=2 | DDX1 |
|  | Q2Q1W2 | Tripartite motif-containing protein 71 OS=Homo sapiens GN=TRIM71 PE=2 SV=1 | TRIM71 |
|  | P26599 | Polypyrimidine tract-binding protein 1 OS=Homo sapiens GN=PTBP1 PE=1 SV=1 | PTBP1 |
|  | P61978 | Heterogeneous nuclear ribonucleoprotein K OS=Homo sapiens GN=HNRNPK PE=1 SV=1 | HNRNPK |
|  | P26368 | Splicing factor U2AF 65 kDa subunit OS=Homo sapiens GN=U2AF2 PE=1 SV=4 | U2AF2 |
|  | Q9UMS4 | Pre-mRNA-processing factor 19 OS=Homo sapiens GN=PRPF19 PE=1 SV=1 | PRPF19 |
|  | Q6P2Q9 | Pre-mRNA-processing-splicing factor 8 OS=Homo sapiens GN=PRPF8 PE=1 SV=2 | PRPF8 |
|  | Q5JW30 | Staufen, RNA binding protein, homolog 1 (Drosophila) OS=Homo sapiens GN=STAU1 PE=2 SV=1 | STAU1 |
|  | Q9UHX1 | Poly(U)-binding-splicing factor PUF60 OS=Homo sapiens GN=PUF60 PE=1 SV=1 | PUF60 |
|  | A6NIT8 | Heterogeneous nuclear ribonucleoprotein L OS=Homo sapiens GN=HNRNPL PE=2 SV=1 | HNRNPL |
|  | F5GYZ3 | Non-POU domain-containing octamer-binding protein OS=Homo sapiens GN=NONO PE=4 SV=1 | NONO |
|  | P08621 | U1 small nuclear ribonucleoprotein 70 kDa OS=Homo sapiens GN=SNRNP70 PE=1 SV=2 | SNRNP70 |
|  | P67809 | Nuclease-sensitive element-binding protein 1 OS=Homo sapiens GN=YBX1 PE=1 SV=3 | YBX1 |
|  | Q13838 | Spliceosome RNA helicase DDX39B OS=Homo sapiens GN=DDX39B PE=1 SV=1 | DDX39B |
|  | E7EUY3 | Propionyl-CoA carboxylase beta chain OS=Homo sapiens GN=PCCB PE=4 SV=1 | PCCB |
|  | D6RAF8 | Heterogeneous nuclear ribonucleoprotein D OS=Homo sapiens GN=HNRNPD PE=4 SV=1 | HNRNPD |
|  | D6R9P3 | Heterogeneous nuclear ribonucleoprotein A/B OS=Homo sapiens GN=HNRNPAB PE=4 SV=1 | HNRNPAB |
|  | Q52LJ0 | Protein FAM98B OS=Homo sapiens GN=FAM98B PE=1 SV=1 | FAM98B |
|  | P26196 | Probable ATP-dependent RNA helicase DDX6 OS=Homo sapiens GN=DDX6 PE=1 SV=2 | DDX6 |
|  | P22626 | Heterogeneous nuclear ribonucleoproteins A2/B1 OS=Homo sapiens GN=HNRNPA2B1 PE=1 SV=2 | HNRNPA2B1 |
|  | Q13151 | Heterogeneous nuclear ribonucleoprotein A0 OS=Homo sapiens GN=HNRNPA0 PE=1 SV=1 | HNRNPA0 |
|  | P14678 | Small nuclear ribonucleoprotein-associated proteins B and B' OS=Homo sapiens GN=SNRPB PE=1 SV=2 | SNRPB |
|  | O75533 | Splicing factor 3B subunit 1 OS=Homo sapiens GN=SF3B1 PE=1 SV=3 | SP3B1 |
|  | Q1KMD3 | Heterogeneous nuclear ribonucleoprotein U-like protein 2 OS=Homo sapiens GN=HNRNPUL2 PE=1 SV=1 | SNRPBUL2 |
|  | Q15393 | Splicing factor 3B subunit 3 OS=Homo sapiens GN=SF3B3 PE=1 SV=4 | SF3B3 |
|  | P22087 | rRNA 2'-O-methyltransferase fibrillarin OS=Homo sapiens GN=FBL PE=1 SV=2 | FBL |
|  | P11940 | Polyadenylate-binding protein 1 OS=Homo sapiens GN=PABPC1 PE=1 SV=2 | PABPC1 |
| rRNA prcessing | Q15393 | Splicing factor 3B subunit 3 OS=Homo sapiens GN=SF3B3 PE=1 SV=4 | SF3B3 |
| Protein biosynthesis | P49411 | Elongation factor Tu, mitochondrial OS=Homo sapiens GN=TUFM PE=1 SV=2 | TUFM |
|  | P68104 | Elongation factor 1-alpha 1 OS=Homo sapiens GN=EEF1A1 PE=1 SV=1 | EEF1A1 |
